# Pancreatic Adenocarcinoma Invasiveness and the Tumor Microenvironment: From Biology to Clinical Trials

**DOI:** 10.3390/biomedicines8100401

**Published:** 2020-10-09

**Authors:** Isabel Mejia, Sandhya Bodapati, Kathryn T. Chen, Begoña Díaz

**Affiliations:** 1 Department of Medicine, Division of Medical Hematology Oncology, The Lundquist Institute for Biomedical Innovation at Harbor-UCLA Medical Center, Torrance, CA 90502, USA; isabel.mejia@lundquist.org; 2 College of Osteopathic Medicine, Pacific Western University of Health Sciences, Pomona, CA 91766, USA; sandhya.bodapati@westernu.edu; 3 Department of Surgery, Division of Surgical Oncology, Harbor-UCLA Medical Center, Torrance, CA 90502, USA; kathryn.chen@lundquist.org; 4 David Geffen School of Medicine, University of California at Los Angeles, Los Angeles, CA 90095, USA; 5 Jonsson Comprehensive Cancer Center, University of California at Los Angeles, Los Angeles, CA 90095, USA

**Keywords:** PDAC, tumor stroma, tumor microenvironment, cell invasion, invadopodia, clinical trial, cancer vaccine

## Abstract

Pancreatic adenocarcinoma (PDAC) originates in the glandular compartment of the exocrine pancreas. Histologically, PDAC tumors are characterized by a parenchyma that is embedded in a particularly prominent stromal component or desmoplastic stroma. The unique characteristics of the desmoplastic stroma shape the microenvironment of PDAC and modulate the reciprocal interactions between cancer and stromal cells in ways that have profound effects in the pathophysiology and treatment of this disease. Here, we review some of the most recent findings regarding the regulation of PDAC cell invasion by the unique microenvironment of this tumor, and how new knowledge is being translated into novel therapeutic approaches.

## 1. Introduction

From an anatomic standpoint, most tissues, including glandular tissues, can be divided into parenchyma (the functional cellular components of the tissue, such as secretory glandular epithelial cells) and stroma (the “supportive” part of the tissue, containing stromal cells and extracellular matrix, blood vessels, nerves and immune cells among others). Parenchymal cells are also exposed to extracellular conditions such as nutrients, pH and oxygen, which along with the stroma, are usually referred to as the tissue microenvironment.

Reciprocal interactions between parenchyma and stroma are essential during embryonic development, normal physiology, and in injury repair [1,2,3,4]. These occur in the form of cell-cell and cell-extracellular matrix interactions, with the former happening through direct cell-to-cell contact, or by long range signals such as released growth factors and signaling vesicles (exosomes). Likewise, parenchymal and stromal component of tumors crosstalk during tumor initiation, progression and therapeutic response. In contrast with the well-orchestrated architectural plan of normal healthy tissues, in which stromal-parenchymal interactions are mutually coordinated following a well-defined plan that ensures optimal tissue function [1,2], the interactions between parenchyma and stroma in tumors are aberrant [4], constantly evolving, and strongly defined by the heterogenous and ever changing nature of cancer cells [5,6].

Indeed, a complex network of reciprocal interactions between cancer, stromal cells and the non-cellular components of the tumor microenvironment has been untangled in pancreatic and other tumor types [7,8]. Whereas the specific players involved in the cross-talk between cancer cells and the tumor microenvironment may differ between different cancers types based on the tissue of origin and driver mutations, the elevated degree of complexity of these interactions holds true for all tumor types, and is particularly complex in pancreatic adenocarcinoma where stroma is very abundant. As such, different components of the stroma and/or the stroma at different stages of tumor progression may be either restrictive of supportive of tumor growth [9,10], a fact with critical implications in therapy design and resistance.

The normal healthy tissue stroma is generally considered as not-permissive for cancer initiation and progression. For instance, the embryonic environment suppresses the growth of tumor cells [11]. However, the restriction that a normal stroma imposes in tumor growth is limited when the tissue’s homeostasis is altered. Thus, inflamed or damaged tissue facilitates cancer formation, as reflected by the increased propensity to develop pancreatic adenocarcinoma in patients with chronic pancreatitis [12]. One of the first examples demonstrating the impact of tissue damage in tumor progression came from studies in chickens infected with Rous sarcoma virus (RSV), which carries the viral oncogenic form Src (v-Src). Sarcomas induced by RSV infection were restricted to the site of injection in control animals, but would spread beyond the site of injection if animals sustained wounds [13]. Subsequent studies from the same group demonstrated that breast cancer cells growing in three dimensions recover functional acinar polarity after blockade of cell-matrix interactions by treatment with anti-ß1 integrin antibodies [14], fostering the interest in understanding the role of the tumor microenvironment in cancer progression. Over the years many studies have dissected the complex interplay between cancer cells and the cancer-associated stroma. Drawing a detailed map of these interactions in space and time taking into account tumor heterogeneity and evolution will more effectively guide targeted therapies [15].

Heterogeneity happens at different levels in tumors and has a profound impact in tumor evolution and response to therapy [16]. The best studied type of tumor heterogeneity is defined by genetic or epigenetic differences in cancer cell subpopulations within the same tumor. Interestingly, polyclonality is also present in PDAC metastatic lesions in mice [17], reflecting the complexity of both primary and metastatic lesions. Transcriptomic sources of epithelial and stromal heterogeneity have also been defined using single cell RNA sequencing both in precursor lesions such as intraductal papillary mucinous neoplasms [18], and in human PDACs, where different subpopulations of malignant pancreatic ductal cells have been identified [19]. Additional forms of tumor heterogeneity are defined by the tumor microenvironmental conditions, such as those determined by the physical proximity of cancer cells to specific stromal components. For instance, distance of cancer cells from blood vessels creates a gradient of nutrients and oxygen, both of which affect cancer cell behavior, metabolic adaptation, and drug response [20]. Additional factors such as intratumoral pH [21], dynamic blood flow [22], and matrix rigidity [23] are also topologically heterogeneous in the same tumor. In addition, pathological observation of tumors often reveals heterogenous histological types, with a mixture of different degrees of pancreatic intra-epithelial neoplasia (PanIN) lesions or even normal-looking ducts and acini intermingled with adenocarcinoma tissue [24]. The possible influence that this type of heterogeneity has in tumor progression is not well understood. The reciprocal interactions between cancer cells and the tumor microenvironment are profoundly affected by all the aforementioned forms of tumor heterogeneity, and in turn, generate heterogeneity themselves [25]. All this complex interplay affects and is shaped by clinical interventions, and change over time as tumor progresses and evolves, promoting tumor growth and malignancy [16,26].

A deeper understanding of the multilayered complexity governing tumor biology is critical to design and improve therapeutic approaches for pancreatic cancer. Here, we present an overview of the different players that contribute to the complexity of the pancreatic tumor microenvironment, and discuss some of the most recent contributions to our understanding of how the tumor microenvironment regulates cancer invasiveness. We also discuss the most recent therapeutic approaches against PDAC, particularly those designed to disrupt the pancreatic tumor microenvironment.

## 2. Cellular Mechanisms of PDAC Invasion

### 2.1. The Pro-Invasive Phenotype of PDAC Cancer Cells

Malignant transformation of epithelial cells is often associated with phenotypic changes that facilitate the acquisition of pro-invasive traits. For example, cancer cells undergoing epithelial to mesenchymal transition (EMT) display loss of cell-cell contacts and gain of migratory ability that facilitates invasiveness [27]. In mouse models of PDAC, cancer cells undergo EMT early during tumor progression [28] and EMT is associated with chemoresistance [29], as well as with cell plasticity and metastasis [30].

**Figure 1:**
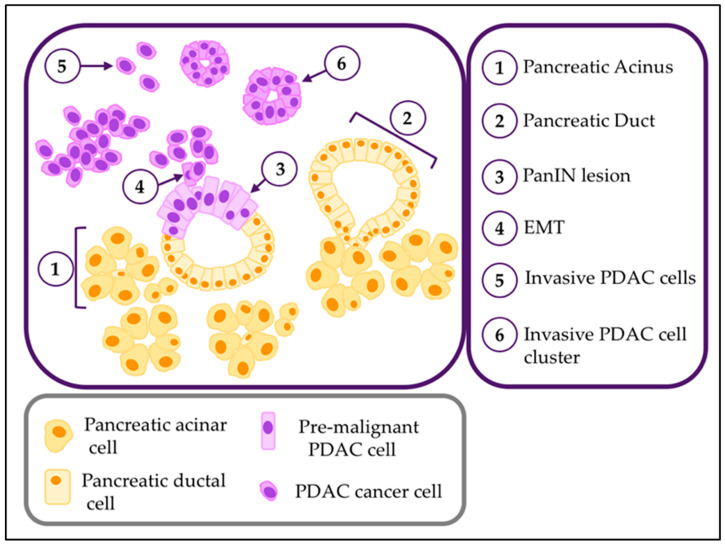
**Normal and PDAC parenchymal cells**. Diagram representing the parenchymal cellular components of the normal exocrine pancreas, PanIN pre-malignant lesions and PDAC. Histological features of each includes acini (1), ducts (2), atypic cells in panIN lesions (3), PDAC cells undergoing epithelial-to-mesenchymal transition (4), invasive PDAC migrating as individual cells.

Cancer cell invasion is a prerequisite for metastasis, and can be defined as the aberrant migration of cancer cells from their original tumor location into surrounding tissues by a process involving cell transit through complex matrix environments. Glandular epithelial cells such as those in the pancreatic exocrine glands are separated from the surrounding stroma by a basement membrane, which is composed of a highly cross-linked mixture of collagens and other matrix proteins, and acts as a boundary between the glandular cells and the underlying pancreatic stroma. The status of the basement membrane (intact *versus* compromised) determines the pathological classification of pancreatic tumors into “in situ” pancreatic intraepithelial neoplasia (PanIN) or “invasive” PDAC (Figure 1). Once the basement membrane is breached, cancer cells are considered to have acquired the ability to proceed through the less cross-linked matrix environment that comprises the tumor stroma, and to reach blood and lymphatic vessels for metastatic dissemination to distant organs. During this process, the tumor microenvironment and its multiple cellular and non-cellular components contribute to modulate (prevent or promote) cancer invasiveness.

It is important to note that acquisition of invasiveness by a small subset of cells within the primary tumor at any given time during tumor evolution is likely enough to shed sufficient cells into the circulation over time so that some of them succeed in completing the process of metastatic colonization. Furthermore, in different experimental models, actively invading cancer cells may be followed by less invasive cells that take advantage of the new route out of the tumor that has been opened by the leading invasive cells [31]. This is in agreement with the collective migration of cancer cells and the presence of circulating tumor cell clusters [32,33]. Indeed, collective migration is observed in surgical-derived human PDAC organoids containing SMAD4 mutations, whereas mesenchymal migration is predominantly observed in PDAC organoids with other mutations [34].

Furthermore, stromal cells may lead the way in tumor invasion, facilitating cancer cells that follow them a route out of the tumor [35]. An improved understanding of the many processes by which cancer cells breach basement membranes and move through the surrounding stroma is important to design of interventions that effectively prevent or limit cancer metastasis.

### 2.2. Invadosome-Mediated Cell Invasion in PDAC

The term “invadosome” collectively refers to protrusive pro-invasive structures named invadopodia (if found in cancer cells) of podosomes (if found in non-cancer cells) [36,37].

**Figure 2:**
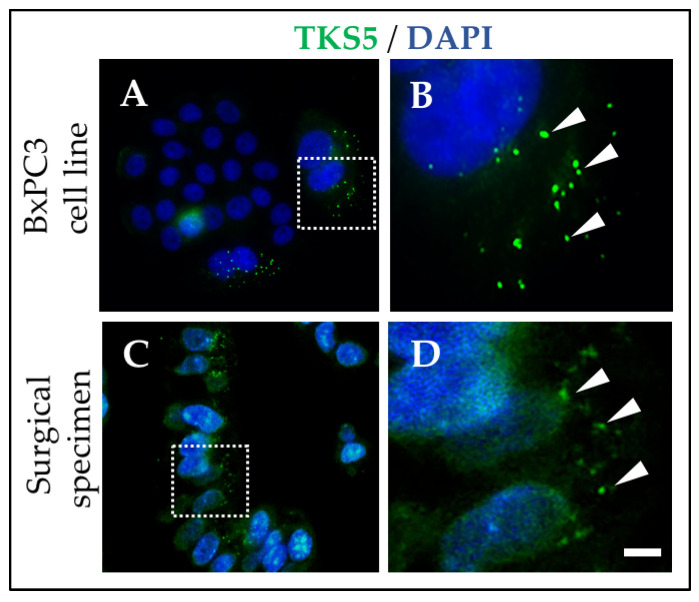
TKS5-positive invadopodia in a PDAC cell line in culture and in a PDAC archived surgical specimen. (**A**) BxPC3 cells were stained with a TKS5 antibody and DAPI. (**B**) Image corresponding to square in A. (**C**) Sections from an archived paraffin-embedded PDAC surgical specimen stained with a TKS5 antibody and DAPI. (**D**) Image corresponding to square in C. Arrowheads, invadopodia (**B**) and invadopodia-like structures (**D**). Bar, 1 μm in A, C and 0.1 μm (**B**,**D**). See also Refs. [48,49,50].

Invadopodia are sites of proteolytic degradation of the ECM, and represent an important mechanism by which neoplastic cells invade [38]. Invadopodia are rich in filamentous actin and contain proteins involved in actin cytoskeleton organization such as cortactin, WASP family members and cofilin. They also are rich in proteinases such as MT1-MMP [37]. Invadopodia are enriched in the adaptor protein and invadopodia marker tyrosine kinase substrate with five SH3 domains (TKS5) [39], which is necessary for invadopodia formation and activity (pericellular proteolysis) in different cancer cells in culture as well as in animal models [40,41,42,43]. The long TKS5 isoform (TKS5α) is the prominent form found in cancer cells [44,45], and it is associated with malignant transformation and with poorer prognosis in several human malignancies including glioblastoma and breast cancer [41,46,47]. TKS5α is expressed in a number of pancreatic adenocarcinoma cell lines, and TKS5-positive invadopodia are elaborated by the pancreatic cancer cells lines BxPC3 and PANC1 [48,49,50] (Figure 2). PDAC cells depleted of TKS5 fail to elaborate invadopodia and degrade gelatin substrates [48].

Cells use invadopodia to cross the basement membranes of intact peritoneal rat membranes in vitro [51]. Furthermore, invadosome-like structures mediate cell invasion during nematode vulvar development [52,53], and cancer cell intravasation in an ex-vivo avian embryo model [43]. Collectively, these and other findings, support a role for invadopodia in cancer invasiveness and metastatic potential in vivo, and indicate that invadopodia are likely used by cancer cells inside tumors to cross the basement membrane, invade through the stroma and enter the circulation. Consistent with this hypothesis, a subset of TKS5 positive cells is found associated with the leading edge in human pancreatic adenocarcinoma surgical specimens [48]. A closer look to TKS5-stained cells in pancreatic surgical specimens revealed the presence of invadopodia-like punctate structures, further suggesting that invadopodia are elaborated by cancer cells, including PDAC, inside human tumors [48,49,50].

Three-dimensional cell culture models in combination with advanced microscopic techniques allow a detailed visualization of cells invading in environments mimicking the tumor stromal architecture. For instance, breast cancer cells embedded in fibrillar type I collagen elaborate TKS5-positive curved invadopodia along collagen fibers, which display membrane type I metalloproteinase (MT1-MMP) dependent collagenolytic activity [54]. MT1-MMP proteolysis opens pores in the collagen matrix, which are further widened by actin polymerization-based forces at expanding circular invadopodia [54]. This study indicates that invadopodia facilitate invasion through collagen fibers by using a combination of proteolytic and mechanical means. This is in agreement with mechanobiological studies showing a correlation between proteolytic degradation and mechanical forces exerted by head and neck cancer cell invadopodia in vitro [55]. Interestingly, some tumoral stromas, such as those found in breast tumors, exhibit high viscosity and plasticity, which can be experimentally mimicked using hydrogels. During migration through these environments, breast cancer extend invadopodia that mechanically deform hydrogel components to open pores through which migrate in a proteolysis-independent manner [56]. Thus, invadopodia may mediate proteolytic-dependent and independent models of invasion. Whereas highly plastic and viscoelastic properties are not found in the relatively rigid environment of basement membranes, they may be present in the underlying stroma of other tumors types. In principle, one would argue that this mechanism of invasion alone would not be efficient in the dense stroma of PDAC. It will be interesting to determine whether or not PDAC cells utilize a mechanism that combines proteolysis and mechanical separation of collagen fibrils when invading through matrices mimicking the desmoplastic stroma.

Collectively, these recent findings suggest that invadopodia are more versatile than previously recognized, because they not only focally degrade the extracellular matrix, but also exert mechanical forces that facilitate the invasion of cancer cells. Furthermore, invadopodia facilitate cancer cell proliferation in 3D collagen matrix [41,42], exosome secretion [57,58] and chemosensing [59]. The latter is mediated by the presence of GABA and EGF receptors at invadopodia, which mediate chemotaxis-induced intravasation of breast cancer cells through a PAK1-dependent mechanism, which in turn promotes metastatic brain tropism [59].

Whereas, by definition, invadopodia are elaborated by cancer cells, podosomes are also found in many stromal cell components in both the normal and the cancer-associated stroma. Thus, invadosome formation and activity is at the crossroads of invasiveness modulated by the cross-talk between cancer cells and the tumor microenvironment [60]. Whether selective inhibition of invadopodia formation for therapeutic purposes can be achieved is still a matter of debate. Because podosome formation by stromal cells might restrain cancer progression, it is important to improve our understanding of these structures and their coordinated function inside tumors to selectively inhibit pro-tumorigenic invadosomes while preserving the function of podosomes that might restrain cancer progression.

## 3. The Tumor Microenvironment and the Regulation of PDAC Invasiveness

This section will discuss how cellular and non-cellular components of the PDAC tumor microenvironment contribute to cancer invasiveness.

### 3.1. Cellular Components of the Pancreatic Adenocarcinoma Microenvironment

**Figure 3:**
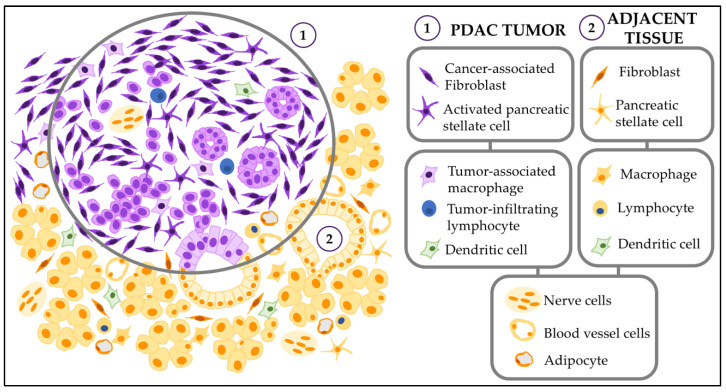
Cellular components of the PDAC tumor microenvironment. Diagram of the cellular components of the PDAC tumor microenvironment (1) and adjacent tissue stroma (2). Tumor cells include tumor-associated stromal cells (left upper box); immune-associated compartment (left middle box); and additional cells (bottom box) such as nerve cells, blood and lymphatic vessels cells, and adipocytes. The normal adjacent tissue stroma contains normal stromal cells (upper right box) and immune normal cells (right middle box). Nerve, blood and fat cells are also present in the adjacent stroma (bottom box).

We will discuss how the cells that constitute the tumor microenvironment affect PDAC invasiveness. Figure 3 summarizes the content of this section.

#### 3.1.1. Cancer Associated Fibroblasts

Cancer associated fibroblasts (CAFs) are the most abundant cell type in the PDAC tumor microenvironment [61]. CAFs are the primary source for the components of the ECM that make up the desmoplastic stroma [61]. These cells are heterogenous populations that can be identified as inflammatory CAFs or myofibroblast CAFs [9,61,62], with the latter expressing αSMA, ECM components, and contractility factors [62]. Inflammatory CAFs express cytokines and chemokines such as IL-6 and CXCL12 [62]. Additionally, there are sets of CAFs that express the MHC II complex and present antigen to T cells [62]. Some CAFs can have gene expression profiles that are not exclusively inflammatory or myofibroblast-like [63], further indicating the plasticity and heterogeneity of this cell populatio [9,10]. Indeed, evidences for tumor-promoting and tumor-inhibitory functions of CAFs in PDAC have been found [9,10], and a consensus statement recently made available highlights the complexity of this tumor component, and makes important technical recommendations to rigorously advance this research field [64].

The cross-talk between CAFs and PDAC also increases the population of self-renewing stem like cells, which sustain tumor growth and exhibit enhanced migratory ability that facilitates invasion and metastasis [65]. For instance, co-culturing PDAC with CAFs promotes cancer stem cell phenotype in those cancer cells in the vicinity of CAFs via induction of integrin-FAK signaling that is essential for cancer stem cell (CSC) regulation [66,67]. In a colorectal adenocarcinoma model, CD44 expressing CAFs, promoted by a hypovascular environment are deemed necessary for the maintenance of CSCs via an undescribed mechanism [68]. One may speculate that the hypovascular stroma of PDAC may promote the expression of CD44 on CAFs to maintain cancer cell stemness. CSCs are supported by CAFs in breast cancer through NF-κB mediated secretion of IL-6 and IL-8 [69]. In a colon cancer model, CAFs can secrete factors to activate the Wnt-β catenin pathway in cancer cells to support cancer stemness, and are able to revert differentiated cells back into CSCs through the Wnt-β catenin pathway [10,70]. A further mechanism through which CAFs are able to promote stemness, exhibited in prostate cancer lines, is through induction of EMT via MMPs [71]. Further investigation is needed to elucidate the role of CAFs in PDAC stemness.

CAFs are recruited by cancer cells through several mechanisms including sonic hedgehog (HH) secretion [72], and several in vitro studies indicate that they promote PDAC invasiveness. For instance, CAFs secrete collagen and lysyl oxidase (LOX) that increases matrix stiffness, which may promote invasion [73]. Remodeling of the ECM by CAFs leads to fibronectin alignment that promotes directional cancer migration [74]. Interestingly, PDAC CAFs themselves elaborate invadosomes through a mechanism involving palladin-dependent Cdc42 activation and remodeling of the ECM that in turn facilitates growth and metastasis of xenografted pancreatic cancer cells [75]. Despite the pro-tumorigenic and pro-invasive role of CAFs in these and additional studies [7], genetic depletion of CAFs in a mouse model of PDAC accelerated disease progression [76]. Likewise, targeting HH signaling, which depletes the stroma of HH-dependent CAFs, enhanced PDAC progression [77,78]. Indeed, depletion of the collagen-rich stromal component of PDACs by targeting lysyl oxidase-like 2 is sufficient to accelerate tumor growth in mice [79]. Thus, the desmoplastic stroma plays a dual role in PDAC progression, and further investigation is needed to better understand the extremely complex cancer-stroma interaction.

#### 3.1.2. Pancreatic Stellate Cells

Pancreatic stellate cells (PSCs) in a quiescent state are rich in lipid droplets containing vitamin A and albumin. In PDAC, PSCs assume the activated state, which involves the loss of the lipid droplets and increased expression of α smooth muscle actin (α-SMA) and desmin [80]. Activated PSCs take on a morphology that more closely resembles myofibroblasts [81], and contribute to the desmoplastic response in the ECM through the secretion of collagen, fibronectin and laminin, and the secretion of MMP2, MMP9, and MMP13, which remodel the ECM [80]. In co-cultures of PSCs and pancreatic cancer cells, the frequency and distance of invasion increases when compared to mono-cultures of pancreatic cancer cell [82]. Pancreatic cancer cells growing as spheroids in the presence of PSCs exhibit increased invadopodia formation and ECM remodeling [83]. In addition, PSCs may lead invasion of PDAC cells by remodeling the ECM via collagen fibers to create a pathway for cancer cells to follow [82], and promote basement membrane destruction in PDAC organoid co-cultures through a mechanism involving the secretion of metalloproteinases by PSCs [84].

Pancreatic cancer cells also attract PSCs via the sonic hedgehog ligand (SHH), which promote transcription of IL-6 by PSCs to modulate the conversion of pre-invasive lesions in the pancreas to invasive tumors [80]. Extracellular signal-regulated kinase 1/2 (ERK1/2) activation is elevated in PSCs from PDAC when compared with PSCs from normal tissue, and inhibition of ERK1/2 in PSCs reduced EMT in pancreatic cancer cells and suppressed cancer-stromal interactions and metastasis [85]. PSCs also increase EMT and invasiveness of pancreatic cancer cell through the HGF/c-Met/survivin pathway [86] and through secreted LIF [87]. Finally, PSCs support PDAC immune evasion through upregulation of IP-10 when co-cultured with pancreatic cancer cells, which increases the infiltration of Foxp3^+^ Tregs while reducing cytoxicity from T-cells and NK-cells [88,89]. Collective, these and other finding indicate that activated PSCs contribute to PDAC progression, and may represent a therapeutic target in PDAC [90].

#### 3.1.3. Immune Stromal Cells

The immune system exerts tumor suppressing activities early during tumor initiation through various immune cells. One mechanism through which the immune system exerts tumor suppression is via effector T cells and dendritic cell function. Dendritic cells are able to internalize antigens from the tumor and process them to present them to CD8^+^ cytotoxic T cells [91]. In turn, the CD8^+^ T cells are able to then recognize and exert cytotoxic effects on the cancer cell [91]. The stroma of PDAC, however, has been shown to impede the functioning of these cells. Dendritic cells are inhibited by tumor secreted factors such as IL-6, IL-10, TGF-β, and GM-CSF [92,93]. There is poor infiltration of CD8^+^ T cells into PDAC tumors, which may be explained by a physical barrier produced by the highly fibrotic dense stroma [92]. Additionally, PDAC tumors are primarily infiltrated by γδ T-cells expressing high levels of T cell exhaustion ligands to inhibit activation of CD4^+^ and CD8^+^ T cells [94]. PDAC tumor cells evade this mechanism of suppression via upregulation of PD-L1 to suppress T-cells and downregulation of MHC Class I to evade T cell recognition [92,95]. In addition to evading the cytotoxic effects of CD8^+^ T cells, PDAC tumors cells drive CD4^+^ T cells to differentiate into the inflammatory T_H_2 subtype [92,96].

Another cell that is able to exert an anti-tumor response is the natural killer (NK) cell. These cells are equipped to recognize and kill cells that are either malignant or infected with virus [97]. However, PDAC is able “educate” NK cells to prevent them from exerting their cytotoxic function [98,99]. PDAC cells may impair NK cell function through the secretion of extracellular vesicles containing integrins and immune regulatory factors which may promote the anergy in NK cells to prevent cytotoxic function [97,99].

The PDAC tumor also exploits existing immune cells to promote an immunosuppresive environment [100]. The PDAC tumor microenvironment is typically infiltrated by suppressive immune cells such as regulatory T cells (T_reg_), myeloid-derived suppressor cells (MDSCs), N2 neutrophils, and tumor-associated macrophages (TAMs) rather than anti-tumor promoting immune cells [98,101]. CD8^+^ T cells, natural killer (NK) cells, and dendritic cells, in contrast, are typically exhausted as the tumor progresses and are unable to exert anti-tumorigenic effects that they may been able to exert during early progression of PDAC [98].

T_regs_ are abundant in PDAC when compared to other T cell populations [102]. PDAC cancer cells upregulate chemokine receptors that are important for the homing of T_regs_ into the tumor [92]. T_regs_ normally function to suppress self-recognition which can cause autoimmunity [102]. However, this function could promote immune evasion through tolerance of PDAC tumors which very closely resemble “self” In PDAC, T_regs_ have been associated with higher incidence of metastasis but the mechanism through which this occurs has not been very well studied in PDAC [102]. Interestingly, the depletion of T_regs_ does not halt tumor invasion. Instead, the other stromal components are reprogrammed to further promote invasion [102]. This highlights a complex role for T_regs_ within the PDAC stroma.

MDSCs support PDAC immune evasion through the suppression of T cells by multiple mechanisms such as TGFβ secretion, nitric oxide and reactive oxygen species generation, and L-arginine depletion [103]. MDSCs also establish a pre-metastatic niche, enhance tumor invasion, and stimulate angiogenesis. In breast cancer models, recruitment of MDSCs into the tumors is reliant on mTOR signaling regulating G-CSF [104], which mediates cross-talk between tumor initiating cells (TIC) that produce G-CSF and MDSC which enhance TIC features to further promote cancer initiation [104]. Depletion of MDSCs activates CD8^+^ T cells that were once suppressed by the MDSCs [103].

Neutrophils that exist in the PDAC stroma are polarized to the tumor promoting N2 state [103]. This N2 polarization is typically characterized by secretion and production of immune suppressive factors such as IL-10, CCL2, and arginase [105]. The presence of CXCR2^+^ neutrophils in PDAC are correlated with poor prognosis [106]. Neutrophils can modulate the stroma to promote tumor invasion via secretion of proteases such as elastase, cathepsin, and MMPs which promote angiogenesis and EMT and increase the migratory ability of PDAC cells [103].

Tumor-associated macrophages (TAMs) in the PDAC stroma are typically polarized to the M2 subtype which has a tumor promoting phenotype [103]. Monocytes are recruited into the PDAC stroma via CCL2 that is secreted by tumor cells [103]. Once recruited, monocytes differentiate into these immunosuppressive, macrophages that support the PDAC tumor in various ways. M2 macrophages are able to provide immune suppression through the secretion of anti-inflammatory cytokines such as IL-10, IL-4, and IL-13 and through expressed receptors such mannose and scavenger receptors [107]. This aids in tumor progression because inflammatory signals are not given to recruit immune cells that may promote anti-tumor effects [107]. TAMs in PDAC can also promote invasiveness through microsomal prostaglandin E synthase-1 and 5-lipoxygenase production which inhibit NK cells and T cells from exerting anti-tumor functions [108]. TAMs also secrete exosomes containing microRNAs (miRNA), such as miR-501-3P, that downregulate TGFBR3 which promotes tumor migration and invasion via the TGF-β signaling pathway [109]. In addition, TAMs are able to promote the remodeling of the ECM and angiogenesis to allow for invasion and metastasis [103]. One mechanism of this action is through the secretion of granulin which activates hepatic stellate cells to produce fibrosis to support the establishment of the metastatic niche in the liver, a common site of metastasis for PDAC [110]. Increases of macrophages in the stroma are also associated with increased amounts of fibrosis, whether this is directly due to signaling from the macrophages is unclear [111]. TAMs further support the tumor microenvironment through the secretion of MMP-9 to promote angiogenesis and further tumor progression [112].

Less well studied, B cells may also contribute to PDAC progression. B cells, like other immune cells, suppress CD8^+^ T cells [103]. B cells may promote tumorigenesis and proliferation of pancreatic cancer [103,113]. However, recent studies show that B cells may be immunostimulatory rather than immunosuppresive [114]. These contradictory functions for B cells in PDAC could suggest a dynamic role within the tumor much like other stromal components. It would be advantageous for further investigation into the mechanisms that promote B cells to be tumor promoting or suppressive.

Mast cells are a less commonly mentioned cell in the tumor microenvironment but have also been shown to promote invasiveness of PDAC and are associated with worse prognosis [115]. Mast cells can promote tumor aggressiveness, angiogenesis, and MMP-dependent invasion [103]. In addition, cross-talk between PSCs and mast cells has been observed in which they promote the activation and proliferation of each other [103]. Mast cell IL-13 and tryptase promotes the proliferation of PSCs [116]. In turn, PSCs, along with PDAC cells, can activate mast cells [116].

TAMs, dendritic cells, and T cells all have the ability to elaborate podosomes [117,118,119,120], which we speculate may either promote or suppress cancer progression. A better characterization of the regulatory signals that control podosome formation and an improved understanding of their contribution to cancer progression is necessary to specifically target invadopodia and pro-tumorigenic podosomes formation while preserving anti-tumorigenic podosomes.

#### 3.1.4. Other Tumor Stromal Cells

Perineural invasion, in which cancer cells invade and move along nerves within tumors, is prominent in PDAC, where it causes significant pain and correlates with decreased patient survival [121]. Ablation of sensory neurons in pancreatic cancer animal models impairs cancer progression, indicating that the interaction between sensory neurons and cancer cells happens before perineural invasion is stablished [121,122]. Additionally, cross-talk between cancer cells and Schwann cells, cells supporting neural cells, has also been described. Cancer cells secrete factors such as NGF, ARTN, CXCL12/SDF-1 which attract Schwann cells, and Schwann cells secrete GDNF, TGF-β and provide NCAM-1-mediated cell-cell interaction to promote cancer cell migration and aggressiveness [123,124]. Close interaction with Schwann cells, therefore, promotes cancer cell invasiveness [121].

Adipocytes in ovarian and breast cancer promote metastasis by acting as an energy source, providing inflammatory factors, and modulating the immune system [125,126]. Interestingly, the PDAC stroma in obese mice is rich in adipocytes, which contribute to the desmoplastic response through crosstalk with PSCs and recruitment of Ly6G^+^ tumor-associated neutrophils [127]. The inflammatory state caused by adipocytes in obese mice also promotes invasiveness of PDAC [127], indicating that stromal adipocytes contribute to the malignancy of PDAC.

Tumor stromal endothelial cells are essential for the formation of the tumor vasculature, which provides nutrients and oxygen, as well as a potential route for metastasis. The invasive edge of PDAC is generally more vascular than the rest of the tumor, suggesting a crucial role of vascularization in invasion [128]. The hypovascularity observed in the rest of the tumor may be explained in part by the fact that PDAC cells induce apoptosis of the endothelial cells that line the lumen of vessels [129]. The invasion of PDAC into vessels can be promoted by tight junctions that are loosened by secreted VEGF [130]. Furthermore, cancer cell exosomes may be uptaken by endothelial cells, and induce angiogenesis and vascular permeability to promote local invasion [131,132], and facilitate vascular permeability in the pre-metastatic niche [128]. Tumor endothelial cells may also promote immune suppression through expression of the junctional adhesion molecule-A (JAM-A) at their tight junctions, thus preventing the infiltration of dendritic cells into the tumor [133].

Pericytes, much like endothelial cells, are associated with the vasculature and act as mural cells for blood vessels [103]. PDAC pericytes are recruited by tumor cells via secretion of vascular endothelial growth factor (VEGF) and platelet-derived growth factor (PDGF) to support angiogenesis [134], which may contribute to the vascularization of the invasive front of the tumor. In other tumor types, pericytes may also support tumor invasion by undergoing a pericyte to fibroblast transition in which pericytes acquire a more fibroblast morphology and contribute to the CAF population within the tumor stroma to facilitate invasion and metastasis [135]. However, pericytes can also have a protective effect by lining the tumor vessels to prevent cancer cells from further invasion and metastasis [135]. PDAC vessels have low levels of pericyte coverage, which may be augmented by inhibition of nucleolin, which in turn decreases proliferation and invasion in PDAC [136].

### 3.2. Non-Cellular Components of the Pancreatic Adenocarcinoma Microenvironment

Here we discuss how the non-cellular components of the tumor microenvironment (extracellular matrix, oxygen and pH) affect PDAC invasiveness.

#### 3.2.1. The Extracellular Matrix (ECM)

The tumor ECM has a profound impact in cancer progression [137]. The ECM proteome or “matrisome” of tumors is comprised of a variety of proteins, including collagens, proteoglycans, proteases, and secreted factors, all of which interact in different ways with both cancer and stroma cells. The complexity of these interactions is highlighted by the fact that, despite increasing vascular normalization and drug delivery [72], targeting the SHH pathway to disrupt the stroma in PDAC accelerated tumor growth [78] and, unfortunately, failed in clinical trials, as detailed later [138].

Indeed, recent findings indicate that the presence of a low stromal component (cellular and acellular) predicts poor prognosis in primary and metastatic human PDAC tumors, indicating a general protective role for the stroma in PDAC [79]. As mentioned earlier, targeting the collagen-rich stroma accelerated PDX orthotopic growth, demonstrating the protective role of the collagen-I rich fibrotic response in PDAC progression [79]. Recent efforts at mapping ECM signatures from tumors using advanced proteomic technologies have improved the understanding of their role in cancer progression [139]. Whereas most ECM proteins are secreted by the pancreatic tumor stromal cells, a small subset is secreted by PDAC cells [140]. Interestingly, PDAC-derived matrisomal components such as serpin B5 and cystatin B promote metastasis through invadopodia formation and intravasation in AsPC1 pancreatic cancer cells [141]. These findings suggest that strategies that selectively target cancer-cell derived stromal components may have therapeutic benefit in PDAC.

Stromal rigidity affects invadopodia formation [142], which in vitro exhibits an optimal range below or above which invadopodia do not efficiently assemble [143]. It would be interesting to understand whether and how the specific machanoproperties of the fibrotic desmoplastic stroma of PDAC affect invadopodia formation. The ECM is also a reservoir for latent growth factors such as TGF-β, which is activated by MT1-MMP-dependent cleavage [144]. Invadopodia could mediate this cleavage, perhaps promoting podosome formation in endothelial cells in the tumor microenvironment [145]. This is just one example of the multiple soluble factors and cytokines present in the tumor matrisome [146,147], which have potential to promote invadosome formation and promote cancer cell invasiveness.

#### 3.2.2. Oxygen Availability and Extracellular pH

PDAC tumors are regarded as highly hypoxic due to a poorly vascularized stroma, in which the elevated interstitial pressure created by the fibrotic reaction further limits blood perfusion and, thus, oxygen availability. However, direct measurement of hypoxia in PDACs from patients using the pimonidazole probe found that the levels of hypoxia inside human PDAC tumors were comparable to those of other tumor types [148]. Interestingly, this study found a high level of intratumoral heterogeneity in hypoxia distribution, which affected both cancer cells and the stroma [148]. Regardless of its level and extent, hypoxia is present inside PDACs and promotes invasion and malignancy through a number of mechanisms [149]. For instance, hypoxia induces the formation of invadopodia in BxPC3 pancreatic cancer cells through HIF1a-dependent activation of Notch1 and increased expression and activity of ADAM12 which in turn, sheds HB-EGF from the surface of cancer cells under hypoxia to promote invadopodia formation in a non-cell autonomous manner [150]. HIF1α also promotes PDAC invasion through a mechanism involving fascin overexpression [151]. In addition, HIF2α, which also responds to hypoxia, mediates invasiveness and is a poor prognostic factor in PDAC [152]. Because the stromal component of human PDACs is also affected by hypoxia [148], the hypoxia adaptative response is also activated in stromal cells. For instance, hypoxia inhibits the secretion of lumican by stellate cells [153], which in turn creates an ECM environment that promotes cancer progression. It is conceivable that hypoxia could also affect the ability of stromal cells to elaborate podosomes, which in turn would promote aberrant matrix remodeling and invasiveness.

In many tumors, including PDAC, the extracellular microenvironment is more acidic than the normal surrounding stroma due to hypoxia and the generation of metabolic by-products [21]. Indeed, pH-based MRI imaging techniques differentiate PDAC from pancreatitis [154]. Invadopodia formation in fibrosarcoma cells in increased by acidic pH through a mechanism involving p90RSK-dependent phosphorylation of the Na ^+^ /H^+^ exchanger NHE1 under hypoxia [155]. The transmembrane enzyme carbonic anhydrase 9 (CA9) contributes to the acidification of the extracellular environment and is necessary for the formation of invadopodia in fibrosarcoma cells [156]. CA9 is induced in PDAC cells after hypoxia and its inhibition decreases tumor growth [157]. It would be interesting to evaluate the role of CA9 in invadopodia formation and metastasis of PDAC.

**Figure 4:**
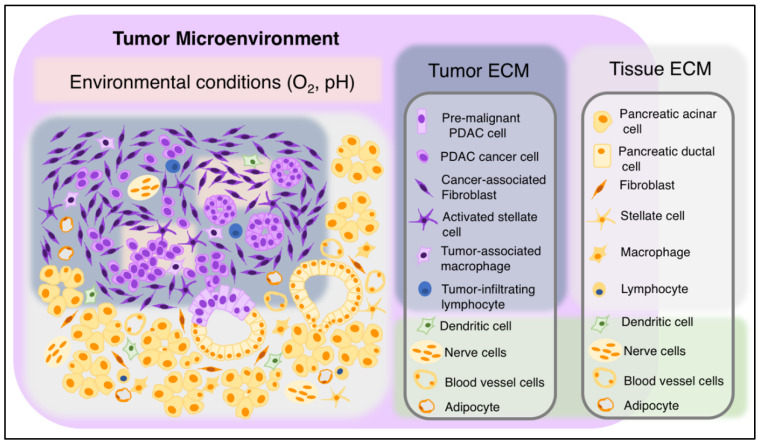
Cellular and non-cellular components of PDAC and adjacent tissue. Left: diagram of PDAC and adjacent tissue components. Right: key to cell identities. Cancer cells and tumor-associated stromal cells (left column) are found in association with adjacent normal epithelial and stromal pancreatic cells (right column). Normal cells are surrounded by tissue ECM (light grey). PDAC cancer and stromal cells are surrounded by the tumor ECM (dark grey). Hypoxia and low pH areas are found inside tumors (light pink). Some normal cells (green shade) can be found inside both tumor and adjacent tumor stroma. Magenta: Tumor microenvironment comprises non-cancer cells, tumor ECM and environmental conditions.

In conclusion, PDAC tumors are composed of diseased cancerous epithelial cells surrounded by a complex array of cancer-associated stromal cells and non-diseased or premalignant epithelial cells. Tumor and normal stromal components intermingle and cross-talk to create multiple complex interactions that affect tumor growth and invasion (Figure 4). Recent advances are shedding light into this complex process, and future studies into the biology of PDAC are warranted to find effective ways to treat PDAC patients.

## 4. Clinical Interventions for PDAC

### 4.1. Current Standard Treatment for PDAC

In 2020, it is estimated that pancreatic ductal adenocarcinoma (PDAC) will comprise 3.2% of new cancer cases and account for 7.8% of cancer deaths, translating to 57,600 new cases and 47,050 deaths in the year (NIH). The overall 5-year survival rate is 9%, with little progress made in the past two decades [158]. Most patients with pancreatic cancer are diagnosed with metastatic disease, and even those patients with seemingly small tumors are thought to harbor micrometastatic disease at the time of presentation [159].

For those patients with potentially curable disease, the mainstay of treatment is typically a combination of chemotherapy and complete surgical resection [160]. Since the 1990s, most chemotherapy regimens have been gemcitabine or 5-fluorouracil (5-FU) based. Both function as inhibitors of DNA replication, resulting in cell death by apoptosis [161,162]. In the past decade, newer combination regimens have come into favor, including FOLFIRINOX (a combination of 5-FU, leucovorin, irinotecan, and oxaliplatin), and gemcitabine with nab-paclitaxel. Combination drugs were created to target multiple points of cell replication [163]. Compared to monotherapy agents, combination regimens have been shown to have measurably improved objective response rates at 23–31%; however, median survival in the metastatic setting remain less than one year [164,165].

Improving objective response rates and overcoming chemoresistance are key issues associated with PDAC treatment, and this is particularly difficult due to the tumor microenvironment (TME) associated with PDAC. Mechanisms of chemoresistance and immune evasion associated with the TME are manifold: there is the general mechanical barrier secondary to desmoplasia and hypoxia which inhibits delivery of drug agents; a high presence of molecules contributing to this dense stroma such as collagen, integrins, glycosaminoglycans (GAGs), and fibronectins; and signaling networks such as platelet-derived growth factor (PDGF), SHH, and many interleukins, which allow for cross-talk between cells of the stroma that contribute to an immunoquiescent and suppressive TME as previously discussed [166,167,168,169].

New translational developments in PDAC treatment that directly address the unique components of the PDAC TME are discussed below and summarized in Table 1 at the end of this part.

### 4.2. Attacking the Dense Fibrous Stroma: FAK and Hyaluronan

As mentioned earlier, the dense fibrous stroma associated with PDAC is one of its defining features, and fibroblastic cells may comprise up to 90% of the pancreatic cancer tumor mass, along with a heterogenous composition of other cell types [170]. Within this nest, tumor cells are protected, as cellular cross-talk within the stromal barrier facilitate resistance to chemotherapy and radiation, and the induced relative hypoxia limits drug delivery efficacy [72,171,172]. Targeting specific stromal components has been attempted to improve delivery and response to chemotherapy and immunotherapy. One classic pathway, SHH, has been extensively studied in pancreatic cancer. Overexpression of ligands associated with this pathway is observed in PDAC epithelial cells, and secretion can activate signaling within the stroma leading to desmoplasia [173,174]. In PDAC mouse models, inhibition of SHH signaling enhanced delivery of gemcitabine, with increased mean tumor vessel density in treated mice [72]. Unfortunately, a recent phase II clinical trial evaluating vismodegib, a SHH inhibitor, in untreated metastatic pancreatic cancer in combination with gemcitabine with nab-paclitaxel failed to demonstrate superiority over historical controls, with a median overall survival of 9.79 months (NCT01088815) [175]. A similar study that preceded this evaluated vismodegib with gemcitabine alone, with similar conclusions [176]. Currently there are no active trials evaluating SHH inhibitors in PDAC.

Focal adhesion kinase (FAK) is overexpressed in PDAC, and leads to increases in type I collagen, fibrosis, and self-renewal of cancer stem cells, as well as inhibition of CD8 anti-tumor T cells [67,177]. FAK became an attractive target to facilitate infiltration of immune cells as well as improve chemotherapy and immunotherapy delivery. In vitro studies conducted in a KPC (Kras, p53, and Cre) PDAC mouse model suggested that treatment by the FAK inhibitor VS-4718 makes previously unreceptive PDAC tumors receptive to immunotherapy and chemotherapy, mainly by depletion of the dense stroma [178]. Initial in vivo research suggested that VS-4718 used in combination with gemcitabine and nab-paclitaxel was effective in treating subcutaneous patient-derived PDAC xenograft tumors in mice [67]. After treatment, relative tumor volume was lowest in the VS-4718, gemcitabine, and nab-paclitaxel group, compared to VS-4718-only treatment group or gemcitabine/nab-paclitaxel-only treatment group [67]. However, FAK inhibitors have yet to be proven in humans and remain in active study. Although there have been several phase I clinical trials with FAK inhibitors, either alone or in combination with chemo- and immunotherapies (NCT00787033, NCT02651727, NCT00666926), there have not been any completed phase II or randomized controlled trials to date. Currently, there are several phase II trials that are accruing patients, including studies evaluating concurrent stereotactic radiotherapy and FAK inhibition (NCT04331041), FAK inhibition with immunotherapy (NCT02758587), and FAK inhibition with immunotherapy following chemotherapy (NCT03727880).

The glycosaminoglycan hyaluronan is another stromal target [179,180]. Hyaluronan has an osmotic effect, such that elevated levels raise interstitial fluid pressure within the tumor mass, inhibiting intratumoral drug delivery due to poor perfusion [180,181,182]. Pegvorhyaluronidase alfa (PEGPH20) was developed as a PEGylated recombinant human hyaluronidase enzyme in order to facilitate depletion of hyaluronan. Mouse models have suggested the efficacy of PEGPH20 in tumors high in hyaluronan [182,183,184]. PEGPH20 was found to inhibit tumor growth by up to 70% in mice containing PC3, 4T1, and Mat LyLu xenograft tumor models expressing high levels of hyaluronan (*p* < 0.001) [183]. In fact, tumor growth inhibition by PEGPH20 in mice is strongly correlated with hyaluronan levels (*n* = 14, *p* < 0.001) [184]. When PEGPH20 was combined with gemcitabine, a survival benefit was also observed in these mice, over gemcitabine alone [182].

Unfortunately, these successes have not borne out in clinical trials. In a phase II clinical trial, patients with metastatic PDAC tumors with high hyaluronan levels were treated with PEGPH20 in combination with gemcitabine and nab-paclitaxel (NCT01839487). Patients treated with this combination therapy were found to have improved progression-free survival compared to those treated with gemcitabine and nab-paclitaxel alone (9.2 months versus 5.2 months, *p* = 0.048) [180]. However, a subsequent phase III trial (NCT02715804) failed to demonstrate superiority of combination PEGPH20 with gemcitabine and nab-paclitaxel over chemotherapy alone (11.2 months versus 11.5 months, *p* = 0.096, *n* = 420) [185]. Currently, there are ongoing trials evaluating PEGPH20 with immunotherapy agents (NCT03634332; NCT03193190); it remains to be seen whether hyaluronan is a viable target.

### 4.3. Cancer Vaccines: GVAX, RAS, Mucins and Kinesins

Vaccines under development are designed to intervene with immune components of the TME, secreted proteins (mucins), as well as proteins expressed by cancer cells which directly or indirectly facilitate PDAC invasiveness (KRAS and kinesins).

#### 4.3.1. GVAX

The PDAC TME also contains a buildup of myeloid-derived suppressor cells (MDSCs), tumor-associated macrophages (TAMs), and regulatory T cells (Tregs), which all downregulate the T-cell immune response [166] as described above. PDAC is known to be immune-quiescent, and immunotherapies, when used as single agents, are generally ineffective, with exceedingly low response rates of 6–17% [186,187]. Vaccine therapies seek to modulate the immunosuppressive TME in order to boost the patient’s innate tumor immune response.

GVAX was developed as a gene transduced pancreatic cancer vaccine that modifies cancer cells to release cytokine granulocyte-macrophage colony stimulating factor (GM-CSF). GM-CSF, in turn, stimulates the differentiation of dendritic cells, which induce an antitumor effect via increased antigen presentation, improved overall effector T cell activity, and induction of macrophages [188,189]. It has been tested in combination with chemotherapy as well as varying immunotherapies, including the immune checkpoint inhibitors programmed cell death protein 1 (PD-1) and cytotoxic T-lymphocyte associated protein 4 (CTLA-4). In murine models, the combination of GVAX and other therapies have proven successful. For instance, treatment by GVAX and a single low dose of DNA alkylating agent cyclophosphamide to lower Treg cell infiltration caused upregulation of PD-L1 expression in mice, and subsequent combination GVAX and anti-PD-1 therapy improved murine survival compared to anti-PD-1 monotherapy (overall survival of 81.5 days versus 50 days, *p* = 0.05) [190]. GM-CSF producing vaccines have been tested along with CTLA-4 blockades in melanoma and lung cancer murine models, yielding promising results of tumor eradication [191].

Preliminary studies in human patients have shown promising, but mixed, results on survival, but have also demonstrated immune responses in some patients. In PDAC patients given intradermal GVAX treatment (*n* = 39), tumors resected two weeks later displayed tertiary lymphoid follicles (comprised of antigen-presenting cells), whereas resected tumors from patients who did not receive the vaccine did not show lymphoid follicles (*n* = 54) (*p* < 0.001) [192,193]. When autologous GM-CSF tumor vaccines were tested in combination with post-resection adjuvant 5-FU-based chemoradiotherapy in a phase II clinical trial with each patient receiving 5 × 10^8^ vaccine cells, median disease-free survival was found to be 17.3 months (NCT00084383) [193], longer than historical controls. Disease-free survival was found to correlate with induction of CD8+ T cells specific to mesothelin epitopes. The same research group also evaluated the efficacy of GVAX in a neoadjuvant approach, with or without cyclophosphamide, followed by adjuvant chemoradiotherapy (NCT00727441). Preliminary results again demonstrated neogenesis of intratumoral tertiary lymphoid aggregates in patients treated with GVAX, and evidence of T-cell response and activation within the TME. They also found that patients who survived over 3 years demonstrated enhanced mesothelin-specific T-cell response with increased PD-1 expression [192]. However, one other phase II study compared GVAX with a live, attenuated Listeria monocytogenes expressing mesothelin against standard chemotherapy failed to show improved survival [194].

The focus of more ongoing studies is evaluating GVAX in combination with immunotherapies, which have been very successful in other cancers such as melanoma. Combination GVAX with ipilimumab, a monoclonal antibody (mAb) targeting immune checkpoint regulation via CTLA-4, was evaluated in a phase II clinical trial (NCT01896869). Patients previously treated with FOLFIRINOX for metastatic PDAC were given combination therapy of GVAX and ipilimumab or allowed to continue on FOLFIRINOX. Overall survival was not improved with GVAX and ipilimumab, although some clinical responses were seen [195]. There are several ongoing studies, including GVAX/cyclophosphamide in combination with anti-PD-1 drug pembrolizumab and stereotactic body radiation therapy (SBRT), a form of targeted radiation (NCT02648282).

#### 4.3.2. Mucins

Mucins (MUCs), O-glycosylated secreted glycoproteins expressed on epithelial cells of organs, have also emerged as promising targets for PDAC vaccine therapy. MUC1, MUC4, MUC5AC, and MUC16 are highly upregulated in the PDAC TME [196]. In fact, MUC4, MUC5AC, and MUC16 are not normally expressed in the pancreas, and are an identifying feature of neoplastic cells [196,197]. Mucin expression is not only associated with pancreatic cancer cell proliferation and metastasis, but also with chemoresistance [196], via activation of anti-apoptotic pathways [198]. In vivo preclinical studies using pan-EGFR inhibitors such as canertinib have modulated EGFR downstream signaling and produced decreases in murine orthotopic pancreatic tumors via MUC4 inhibition [199]. Such studies show promise for further investigation of MUC4 and other mucins for clinical translation in PDAC immunotherapy.

The PANVAC-VF vaccine that targets MUC1, carbinoembryonic antigen (CEA), a serum biomarker elevated in pancreatic cancer, and intracellular adhesion molecule-1 (ICAM-1), and leukocyte function-associated antigen 3 (LFA-3), was tested in combination with GM-CSF in patients with metastatic pancreatic cancer who were unresponsive to gemcitabine therapy (NCT00088660) [200,201]. Unfortunately, the phase III trial did not meet its primary endpoint of improved overall survival [202]. Since then, Cvac, an autologous dendritic cell therapy designed to stimulate immune response against cells with high expression of MUC1, was designed to be tested in a phase II clinical trial as adjuvant therapy for patients who underwent CIS (NCT02310971). The trial was withdrawn by the sponsor, however, due to long approval times. Another clinical trial testing the anti-MUC1 mAb, BTH1704 in combination with Imprime PGG, a glucan from yeast that triggers T cell response, and gemcitabine was terminated early due to drug recall (NCT02132403). More clinical testing is necessary to assess the clinical efficacy of anti-mucin therapy in PDAC.

#### 4.3.3. Oncogenic KRAS

Another cancer vaccine has been developed against the Ras protein antigen, a product of the KRAS gene, which is mutated in the vast majority of PDAC tumors [203,204]. The oncogenic KRAS mutation allows for activation of many signaling pathways and transcription factors that promotes tumor cell proliferation and metastases [205]; silencing of KRAS results in attenuated tumor growth [206]. A long term follow-up on patients enrolled in phase I/II clinical trials who received the ras-peptide cancer vaccine after curative-intent surgery (CIS) showed that 85% of patients had T-cell response to the vaccine [207]. The median overall survival was 28 months vs. 27.4 months for the 85% of patients who responded immunologically to the vaccine (*n* = 23, with 20 evaluable patients) [207]. Though overall survival benefit amongst vaccinated patients was minimal, it is notable that 10-year survival was 20% in those who received the vaccine (*n* = 20) compared to 0% in a cohort that was not vaccinated during the same period (*n* = 87) [207].

Mutant Ras peptide vaccines have been tested in combination with other immunological targets as well. In a phase I/II clinical trial, 58% of patients who received an intradermal synthetic mutant ras peptide vaccine along with GM-CSF elicited an immunological response (*n* = 43) [208]. Furthermore, those who were immunological responders showed improved survival compared to others (148 days versus 61 days, *p* = 0.0002) [208]. Most recently, Targovax developed TG01, a peptide cocktail vaccine that targets 7 peptides mutated in most PDAC tumors, including mutant ras. The phase I trial demonstrated delayed-type hypersensitivity immune response in 100% of patients (*n* = 6). Currently, TG01 is being tested in combination with GM-CSF and gemcitabine in a phase II clinical trial (NCT02261714). KRAS remains an oncogenic target of interest.

#### 4.3.4. Kinesins

Finally, the kinesin superfamily of proteins, specifically Kinesin family member 20A (Kif20A) has emerged as a promising target for PDAC treatment. Kif20a promotes proliferation and metastases of PDAC cells via its role in transport and trafficking of organelles and macromolecules [209]. Silencing of kinesin proteins, including Kif20A has proven to reduce growth of pancreatic cancer in preclinical models [209,210]. A recent phase II trial evaluated the efficacy of the tumor antigen-based peptide cocktail vaccine OCV-C01 derived from antigen Kif20A and vascular endothelial growth factor receptors 1 and 2 (VEGFR1 and VEGFR2), in combination with gemcitabine as adjuvant treatment pancreatectomy (*n* = 30) (UMIN000007991) [211]. The overall disease free survival was 15.8 months for patients treated with OCV-C01 and gemcitabine, and survival was significantly correlated with Kif20A expression in tumor specimens [211,212].

### 4.4. Other Targeted Therapies for PDAC

Lastly, targeted agents against signaling molecules relevant for the crosstalk between PDAC cells and the TME are continually being tested. For example, the colony-stimulating factor 1 receptor (CSF1R) has received attention for its role in promoting the immunosuppressive environment in PDAC by promoting myeloid cell populations, including TAMs and MDSCs [213]. Many preclinical studies have found that blockade of the CSF1R can promote antitumor immunity in the PDAC TME via increased antigen presentation and T-cell response, and upregulation of T-cell checkpoint proteins such as programmed death ligand-1 (PDL-1) and CTLA-4 [213,214]. In fact, cabiralizumab, a CSF1R antagonist, was tested in combination with nivolumab and chemotherapy in a phase II clinical trial (NCT03336216). However, the study ultimately did not meet its primary endpoint of improved progression-free survival compared to chemotherapy alone [215]. Other combination therapy clinical trials are in progress, such as the CSF1R inhibitor pexidartinib with PD-1 inhibitor durvalamab, and another with CSF1R inhibitor IMC-CS4, pembrolizumab, and GVAX/cyclophosphamide (NCT02777710, NCT03153410 respectively).

Connective tissue growth factor (CTGF) or cellular communication network factor 2 (CCN2) is a fibrosis-related gene upregulated in many diseases such as idiopathic pulmonary fibrosis, Duchenne muscular dystrophy, and cancers, including unresectable pancreatic cancer [216]. PDAC cancer cells with upregulated CTGF/CCN2 evade hypoxia-mediated apoptosis [216]. In a phase II clinical trial, 33.3% of patients with initially deemed unresectable PDAC tumors treated with pamrevlumab, a mAb targeting CTGF/CCN2, were able to ultimately undergo surgical resection as compared to 7.7% who received chemotherapy alone (NCT02210559) [217]. Pamrevlumab has received fast-track status by the United States Food and Drug Administration (FDA) for testing as neoadjuvant therapy for unresectable PDAC and is currently undergoing a phase III clinical trial in combination with gemcitabine and nab-paclitaxel (NCT03941093).

As aforementioned, CAFs are key players contributing to the dense fibrous stroma in PDAC tumors. Fibroblast activation protein (FAP) are a surface marker of CAFs, and targeting FAP+ CAFs has been associated with overcoming the immunosuppressive cancer TME [61,218]). FAP+ CAFs secrete CXCL12, which binds to the chemokine receptor 4 (CXCR4) [219], and plays a role in gemcitabine chemoresistance [220]. In a preclinical mouse model, targeting of chemokine ligand 12 (CXCL12) improved antitumor immunity and caused tumor reduction via T cell activity and upregulation of PD-L1 and CTLA-4, highlighting the role of CXCL12 in immune response evasion [219]. In a phase I/II clinical trial, NOX-A12, a CXCL12 inhibitor, was tested in combination with pembrolizumab in metastatic pancreatic cancer and colorectal cancer patients where anti-PD-1 monotherapy was unsuccessful (NCT03168139). Two weeks after the combination treatment, immune response was noted in 50% of the patients (*n* = 11) [221]. Furthermore, stable disease was noted in 25% of patients, and 35% experienced prolonged time on combination treatment compared to prior therapy [221]. CXCL12 thus shows promise as a target for PDAC immunotherapy.

## 5. Conclusions

Despite thousands of experiments and hundreds of clinical trials with many advancements in the scientific understanding of the dense fibrous stroma and immunosuppressive environment of pancreatic cancer, progress in achieving prolonged disease-free and overall survival remain limited. Translation from pre-clinical experiments to human clinical trials is extraordinarily challenging. However, as we develop better understanding of the features of the PDAC TME, and identify strong biomarkers such as invadopodia markers, there is hope for success in future targeted therapies.

## Figures and Tables

**Table 1:** Summary of Clinical Trials targeting the TME and/or cancer cell invasive ability in PDAC.

Pathway (Drug)	Clinical Trial Number	Trial Summary	Status	First Posted	Outcome
FAK inhibition (Defactinib)	NCT02758587	Defactinib concurrent with PD-1 inhibition, Phase I/II	Recruiting	2016	No results posted
	NCT04331041	SBRT^1^ concurrent with defactinib, Phase I/II	Not yet recruiting	2020	
	NCT03727880	Standard peri-operative chemotherapy, with PD-1 inhibition +/− defactinib, Phase II	Recruiting	2018	No results posted
Hedgehog inhibition (GDC-0449, Vismodegib)	NCT01096732	Preoperative administration of GDC-0449, Phase II	Terminated due to slow recruitment	2010	No results posted
	NCT01088815	GDC-0449 in combination with Gem/Abraxane, Phase II	Completed	2010	Addition of GDC-0449 to chemo did not improve efficacy as compared with historical rates with chemotherapy alone [175]
	NCT01064622	Vismodegib with gemcitabine, Phase I/II	Completed	2010	Addition of vismodegib did not improve response rate, OS, or PFS [176]
Hyaluronic acid (PEGPH20)	NCT03634332	Combination PEGPH20 and PD-1 inhibitor for patients with previously treated Hyaluronan-high tumors, phase II	Recruiting	2018	No results posted
	NCT03193190	Combination PEGPH20 and PD-L1 inhibitor for previously treated patients, phase II	Recruiting	2017	No results posted
	NCT02715804	Gem/Abraxane +/− PEGPH20 in patients with hyaluronan-high tumors, phase III	Terminated (Sponsor decision)	2016	PEGPH20 did not improve efficacy of Gem/Abraxane or improve outcomes [185]
	NCT01839487	Gem/Abraxane +/− PEGPH20, Phase II	Completed	2013	Combination therapy with PEGPH20 shows improved progression-free survival (9.2 months versus 5.2 months) [180]
GVAX	NCT00084383	GVAX + adjuvant chemoradiotherapy in resected pancreatic cancer, phase II	Completed	2004	Improved median disease-free survival compared to historical data (17.3 months) [193]
	NCT00727441	GVAX +/− Cy followed by surgical resection and standard adjuvant chemoradiotherapy, phase II	Completed	2008	Not published
	NCT01896869	Ipilimumab/GVAX vs. FOLFIRINOX, phase II	Completed	2013	Ipilimumab/GVAX did not improve OS over continuation of chemotherapy; however clinical responses were observed [195]
	NCT02004262	Cy/GVAX + CRS-207 vs. CRS-207 vs. standard chemotherapy, phase II	Completed	2013	Cy/GVAX + CRS-207 did not improve survival over chemotherapy [194]
	NCT02243371	Cy/GVAX/CRS-207 +/− nivolumab, phase II	Completed	2014	There was no difference in survival between the two arms [222]
	NCT02648282	Cy/Pembrolizumab/GVAX + SBRT	Recruiting	2016	No results posted
RAS vaccine	NCT02261714	TG01/GM-CSF + gemcitabine, phase II	Completed	2014	Improved median OS and DFS (33.1 and 13.9 months) compared to historical data for gemcitabine alone [223]
MUC vaccine	NCT00088660	PANVAC™-VF Vaccine + GM-CSF vs. best supportive care or palliative chemotherapy, phase III	Not recruiting	2004	Did not meet endpoint
	NCT02310971	CVac +/− chemoradiotherapy, phase II	Withdrawn (Sponsor decision)	2014	No results posted
	NCT02132403	IMPRIME PGG^®^ With Anti-MUC1 mAb (BTH1704) and Gemcitabine, phase I	Terminated (Drug recall)	2014	No results posted
Kinesin (Kif20A)	UMIN000007991	Multipeptide cocktail vaccine (OCV-C01) + gemcitabine, phase II	Completed		Favorable median disease-free survival compared to historical data (15.8 months) [211]
CSF1R (Cabiralizumab)	NCT03336216	Cabiralizumab + nivolumab/chemo	Not recruiting	2017	Did not meet endpoint
Connective Tissue Growth Factor (pamrevlumab)	NCT02210559	Pamrevlumab + Gem/nab-paclitaxel vs. Gem/nab-paclitaxel	Not recruiting	2014	Patients who received Pamrevlumab were more likely to undergo resection vs. those who received chemotherapy alone [217]
